# Combretastatin A-4 efficiently inhibits angiogenesis and induces neuronal apoptosis in zebrafish

**DOI:** 10.1038/srep30189

**Published:** 2016-07-25

**Authors:** Yun-Wei Shi, Wei Yuan, Xin Wang, Jie Gong, Shun-Xing Zhu, Lin-Lin Chai, Jia-Ling Qi, Yin-Yin Qin, Yu Gao, Yu-Ling Zhou, Xiao-Le Fan, Chun-Ya Ji, Jia-Yi Wu, Zhi-Wei Wang, Dong Liu

**Affiliations:** 1 Co-innovation Center of Neuroregeneration, Jiangsu Key Laboratory of Neuroregeneration, Nantong University, Nantong, Jiangsu 226001, PRC; 2 School of life science, Nantong University, Nantong, Jiangsu 226001, PRC; 3 Laboratory Animal Center, Nantong University, Nantong, Jiangsu 226001, PRC; 4 School of medicine, Nantong University, Nantong, Jiangsu 226001, PRC; 5 Department of Pharmacology, University of California, Irvine, CA 92697, USA

## Abstract

*Cis*-stilbene combretastatin A-4 (CA-4) and a large group of its derivant compounds have been shown significant anti-angiogenesis activity. However the side effects even the toxicities of these chemicals were not evaluated adequately. The zebrafish model has become an important vertebrate model for evaluating drug effects. The testing of CA-4 on zebrafish is so far lacking and assessment of CA-4 on this model will provide with new insights of understanding the function of CA-4 on angiogenesis, the toxicities and side effects of CA-4. We discovered that 7–9 ng/ml CA-4 treatments resulted in developmental retardation and morphological malformation, and led to potent angiogenic defects in zebrafish embryos. Next, we demonstrated that intraperitoneal injection of 5, 10 and 20 mg/kg CA-4 obviously inhibited vessel plexus formation in regenerated pectoral fins of adult zebrafish. Interestingly, we proved that CA-4 treatment induced significant cell apoptosis in central nervous system of zebrafish embryos and adults. Furthermore, it was demonstrated that the neuronal apoptosis induced by CA-4 treatment was alleviated in p53 mutants. In addition, *notch1a* was up-regulated in CA-4 treated embryos, and inhibition of Notch signaling by DAPT partially rescued the apoptosis in zebrafish central nervous system caused by CA-4.

Current comprehension of solid tumor architecture and microenvironment has led to great progresses in targeting malignant tumor treatments. The tumor vasculature supplies with the requisites for cellular processes, and appears vigorous in the invasion and extravasation of primary tumor cells and eventual metastasis^1^. Therefore, the tumor vasculature is commonly supposed to be target for anticancer therapy. The prominent strategies of targeting tumor vasculature are anti-vasculature and anti-angiogenesis, which target the established tumor vasculature resulting in tumor cell death and prevent the neovascularization in solid tumors respectively^2^. Anti-angiogenesis only is insufficient for achieving effective tumor restraint and the combination therapies in clinical trials is a tendency^3^.

To target tumor vasculature, a large number of vascular disrupting agents (VDAs) have been obtained. As the prototype of many VDAs, *cis*-stilbene combretastatin A-4 (CA-4, Figure S1) showed significant anti-angiogenesis activity, which was firstly isolated from the bark of South-African bush willow *Combretum caffrum*^4^. Following CA-4, a large group of derivant compounds have been achieved, and acquired favorable effects as potential therapeutic candidates for cancer treatment^5^,^6^. In phase II/III clinical trials, the prodrug combretastatin A-4 disodium phosphate (CA-4P, Figure S1) demonstrated favorable efficacy^7^,^8^,^9^. Up to now, it was revealed that CA-4 shuts down the tumor vascular, and inhibits the tumor growth and metastasis through targeting the colchicine-binding site of tubulin in a wide variety of preclinical tumor models^1^,^10^. α-tubulin and β-tubulin heterodimers assemble microtubules, and within tubulin heterodimer, paclitaxel-, vinca alkaloid- and colchicine-binding sites are the major targeting sites^11^. The ligand compounds to tubulin participate in the microtubule dynamics through changing the homeostasis of polymerization and depolymerization of tubulin^12^. Antagonizing tubulin polymerization into microtubules plays a crucial role in the formation of the mitotic spindle and results in cell cycle arrest and apoptosis^2^. So far, the mechanism of anti-vascular of CA-4, with similar molecular skeleton of colchicine (Figure S1), is well documented and concluded as disrupting mitotic spindle by binding to colchicine-binding site in the tubulin dimer^13^. Though great many of VDAs following the prototype CA-4 have been obtained^14^,^15^,^16^, the side effects even the toxicities of these compounds were not evaluated adequately.

The zebrafish model has been becoming an important vertebrate model for assessing drug effects^17^. This model fits into multiple stages of the drug discovery pipeline, from target identification to lead optimization of absorption, distribution, metabolism and excretion, and toxicity studies^18^. The testing of CA-4 on zebrafish model is so far lacking and assessing CA-4 on this model will provide with new insights of understanding the function of CA-4 on angiogenesis, the toxicities and side effects of CA-4.

## Results

### The effects of CA-4 treatment on zebrafish embryos

**Figure 1:**
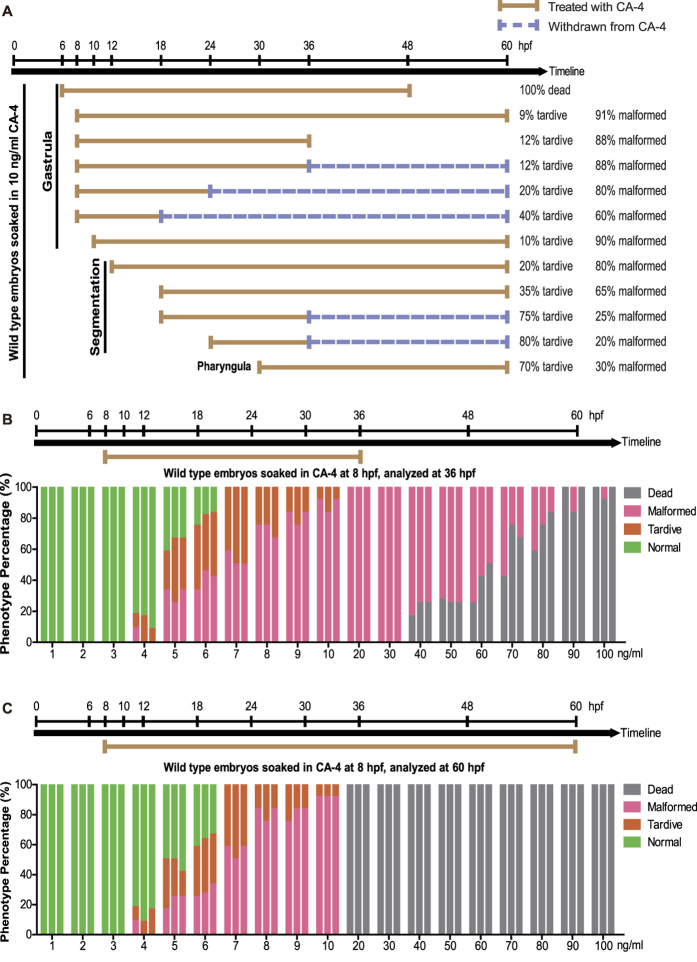
The effects of CA-4 treatment on zebrafish embryos. (**A**) Investigation of the suitable time window of 10 ng/ml CA-4 treatment. The phenotypes of embryo dosed at gastrula, segmentation, pharyngula periods were analyzed. The experiments of CA-4 treatment during each time window were repeated in triplicate at least. (**B,C**) Percentage of phenotype induced by 1–100 ng/ml CA-4 treatment at 8 hpf, analyzed at 36 hpf (**B**) and 60 hpf (**C**), respectively. The experiments of CA-4 treatment at each concentration were repeated in triplicate. The percentage of Dead, Malformed, Tardive and Normal was displayed in Grey, Pink, Orange and Green columns, respectively.

To investigate the suitable dosing time window, we treated zebrafish embryos with 10 ng/ml CA-4 solution at gastrula, segmentation and pharyngula period (Fig. 1A). At 6 hours post-fertilization (hpf) (early gastrula period), 10 ng/ml CA-4 treatment resulted in all embryos dead within 48 hpf. Then we dosed at later periods (from middle gastrula period 8 hpf to pharyngula period 30 hpf). As shown in Fig. 1A, dosing 10 ng/ml CA-4 at different time caused developmental retardation and malformation in different extent. Subsequently, we investigated the dosage of CA-4 between 1 ng/ml and 100 ng/ml, at which embryos were treated in triplicate from 8 hpf (Fig. 1B,C). CA-4 treatment at concentration higher than 40 ng/ml from 8 hpf to 36 hpf caused embryos dead or malformed (Fig. 1B). CA-4 treatment at concentration higher than 20 ng/ml from 8 hpf to 60 hpf resulted in all embryos dead (Fig. 1C). Part of the embryos with CA-4 treatment at concentration lower than 6 ng/ml appeared normal (Fig. 1C). Due to that the CA-4 treatment at the concentration higher than 10 ng/ml caused very severe phenotype, we selected 7, 8 and 9 ng/ml as the working dosages in the following research. The morphology of CA-4 treated embryos was shown in Figure S2.

### CA-4 treatment blocked zebrafish embryonic angiogenesis

**Figure 2:**
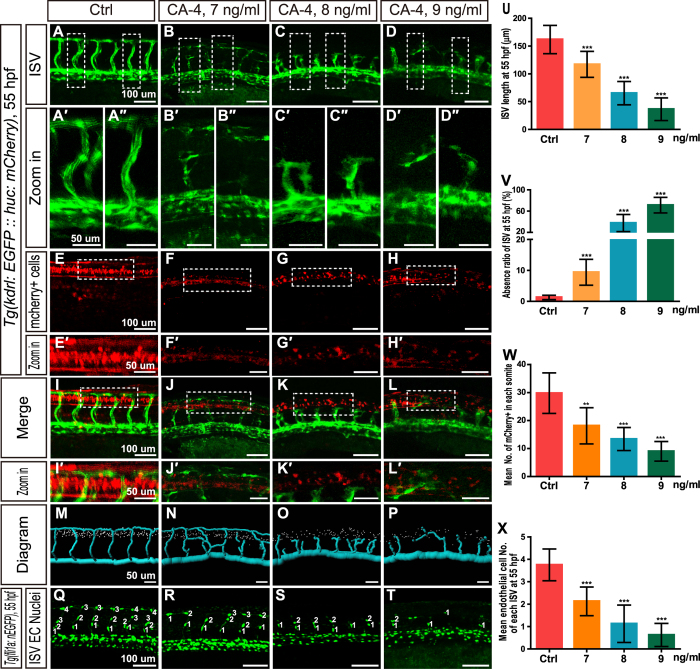
Effects of CA-4 treatment on vascular and central nervous systems in the trunk of *Tg(kdrl:EGFP::huc:mcherry)* and *Tg(fli1a:nEGFP)* zebrafish embryos at 55 hpf. (**A–D**) ISV phenotypes of control group and 7, 8, 9 ng/ml CA-4 treated groups. Scale bar, 100 μm. (A′–D″) Zoomed in images of regions in dash line rectangles of panel (**A–D**). Scale bar, 50 μm. (**E–H**) mCherry positive cells in neural tubes of control group and 7, 8, 9 ng/ml CA-4 treated groups. Scale bar, 100 μm. (E′–H′) Zoomed in images of regions in dash line rectangles of panel E-H. Scale bar, 50 μm. (I–L) Merged images of (A–H). Scale bar, 100 μm. (I′–L′) Zoomed in images of regions in dash line rectangles of panel I-L. Scale bar, 50 μm. (**M–P**) Diagrams of ISVs and neuronal precursor cells in different groups. Scale bar, 50 μm. (**Q–T**) Endothelial nuclei of control group and 7, 8, 9 ng/ml CA-4 treated groups. Scale bar, 100 μm. (**U**–**X**) Statistical analyses of ISV length, ISV absence ratio, number of neuronal precursor cell in each somite, and mean endothelial cell number of each ISV in control group and 7, 8, 9 ng/ml CA-4 treated groups. Data were expressed as the mean ± S.E.M. (n = 8). **P < 0.01 and ***P < 0.001 vs. control group.

**Figure 3:**
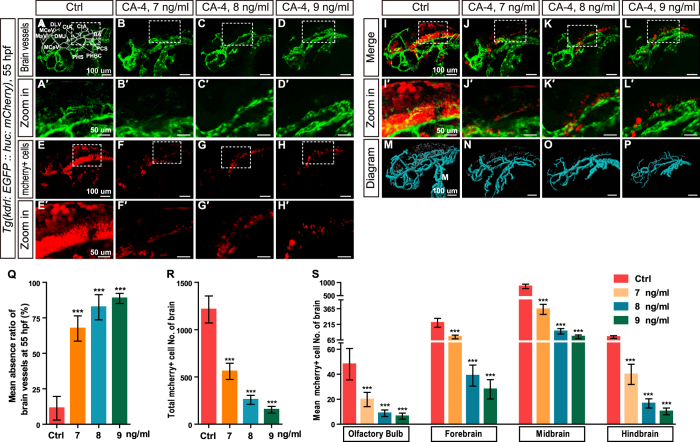
Effects of CA-4 treatment on vascular and central nervous systems in the brain of *Tg(kdrl:EGFP::huc:mcherry)* embryonic zebrafish at 55 hpf. (**A–D**) Phenotypes of brain vessel in control group and 7, 8, 9 ng/ml CA-4 treated groups. Scale bar, 100 μm. Brain vessels dorsal longitudinal vein (DLV), dorsal midline junction (DMJ), middle cerebral vein (MCeV), mesencephalic vein (MsV), central artery (CtA), basilar artery (BA), posterior communicating segment (PCS), primordial hindbrain channel (PHBC), and primary head sinus (PHS) were statistically analyzed. (A′–D′) Zoomed in images of regions in dash line rectangles of panel A–D. Scale bar, 50 μm. (**E–H**) mCherry positive cells in brain of control group and 7, 8, 9 ng/ml CA-4 treated groups. Scale bar, 100 μm. (E′–H′) Zoomed in images of regions in dash line rectangles of panel E-H. Scale bar, 50 μm. (**I–L**) Merged images of (**A–H**). Scale bar, 100 μm. (I′-L′) Zoomed in images of regions in dash line rectangles of panel I-L. Scale bar, 50 μm. (**M–P**) Diagrams of brain vessels and neuronal precursor cells. Scale bar, 100 μm. (**Q–S**) Statistical analyses of mean absence ratio of brain vessels, Number of neuronal precursor cell in brain, Olfactory bulb, forebrain, midbrain and hindbrain in control group and 7, 8, 9 ng/ml CA-4 treated groups. Data were expressed as the mean ± S.E.M. (n = 8). ***P < 0.001 vs. control group.

To verify whether CA-4 blocks embryonic angiogenesis in zebrafish, we investigated the effects of CA-4 using transgenic line *Tg(kdrl:EGFP::huc:mcherry)*, in which endothelial cells (ECs) are labeled in EGFP. It was shown that CA-4 treatment at 7, 8, and 9 ng/ml significantly inhibited ISV branching angiogenesis (Fig. 2A–D,A′-D″). ISV length in the CA-4 treated group was obviously shorter than that of control group (Fig. 2U) and most of the ISV failed to form dorsal longitudinal anastomotic vessel (DLAV) until 55hpf (Fig. 2A–D,A′-D″). The CA-4 treatment caused some ISVs absent especially in 9 ng/ml group (Fig. 2V). Additionally, we found that CA-4 treatment resulted in the brain vessels dorsal longitudinal vein (DLV), dorsal midline junction (DMJ), middle cerebral vein (MCeV), mesencephalic vein (MsV), central artery (CtA), basilar artery (BA), posterior communicating segment (PCS), primordial hindbrain channel (PHBC), and primary head sinus (PHS) partly absent (Fig. 3A–D,A′-D′). The absence ratio was presented as means of ratio of the ten marked brain vessels in CA-4 treated embryos (Fig. 3A,Q). In order to study proliferation and migration of endothelial cells (ECs) in CA-4 treated embryos, the transgenic zebrafish line *Tg*(*fli1a*:*nEGFP*) was employed (Fig. 2Q–T). In control embryos, there were 3–4 ECs in each ISV (Fig. 2Q), however, in the CA-4 treated embryonic zebrafish there were only 1–2 ECs in each ISV, suggesting that proliferation and migration of ECs were significantly inhibited (Fig. 2R–T,X). In addition, we did not observe any significant change of lumen size in DAs or PCVs of CA-4 treated embryos (Figure S3A,B). To examine the arterial-venous differentiation of early ECs in CA-4 treated embryos, we performed whole-mount *in situ* hybridization analysis by using *flt4* and *dll4* antisense probes. The expression of *flt4* in PCV and *dll4* in DA were not apparently changed in CA-4 treated embryos (Figure S3C).

### CA-4 treatment inhibited angiogenesis during fin regeneration in adult zebrafish

**Figure 4:**
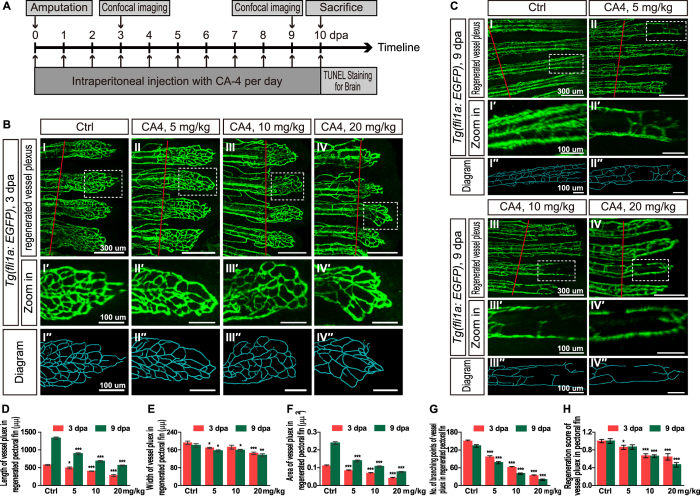
Effects of CA-4 treatment on vessel plexus in regenerated pectoral of *Tg(f li1a:EGFP)* adult zebrafish at 3 dpa and 9 dpa. (**A**) The design and pipeline of the experiments. (BI–IV) Vessel plexus in regenerated pectoral fin of control group and 5, 10, 20 mg/kg CA-4 treated groups at 3 dpa. Scale bar, 300 μm. The amputation sites were marked with red lines. (BI′–IV′) Zoomed in images of regions in dash line rectangles of panel B I–IV. Scale bar, 100 μm. (BI″–IV″) Diagrams of vessel plexus in regenerated pectoral fin generated with Imaris software. Scale bar, 100 μm. (CI–IV) Vessel plexus in regenerated pectoral fin of control group and 5, 10, 20 mg/kg CA-4 treated groups at 9 dpa. Scale bar, 300 μm. The amputation sites were marked with red lines. (CI′–IV′) Zoomed in images of regions in dash line rectangles of panel H-K. Scale bar, 100 μm. (CI′′–IV′′) Diagrams of vessel plexus in regenerated pectoral fin generated with Imaris software. Scale bar, 100 μm. (**D–H**) Statistical analyses of the length, width, area, branching points and regeneration score of vessel plexus in regenerated pectoral fin of control group and 5, 10, 20 mg/kg CA-4 treated groups at 3 and 9 dpa. Data were expressed as mean ± S.E.M. (n = 8). *P < 0.05, **P < 0.01 and ***P < 0.001 vs. control group.

Furthermore, we investigated whether the CA-4 treatment inhibits angiogenesis in adult zebrafish. We measured the length, width and area of regenerated vessel plexus in pectoral fins at 3 days post amputation (dpa) (Fig. 4A,B) and 9 dpa (Fig. 4A,C). It was demonstrated that injection of 5, 10 and 20 mg/kg CA-4 significantly reduced the size of regenerated vessel plexus (Fig. 4B–F). We counted the number of branching points in regenerated vessels and defined the Number of branching points divided by Area of regenerated vessel plexus as regeneration score. It was shown that the number of branching points and the regeneration score of regenerated vessel plexus were significantly reduced in 5–20 mg/kg CA-4 injected zebrafish (Fig. 4G,H). Additionally, the regenerated vessel plexus were diagramed using Imaris software (Fig. 4BI″–IV″).

### CA-4 treatment impairs zebrafish neural development

During investigating the effects of CA-4 on angiogenesis using transgenic line *Tg(kdrl:EGFP::huc:mcherry)*, we found CA-4 treatment at 7–9 ng/ml resulted in significant reduction of number of mCherry positive cells in spinal cord (Fig. 2E–H,E′–H′) and brain (Fig. 3E–H,E′–H′). The number of mCherry positive cells was diagrammed and counted using Imaris software (Figs 2M–P and 3M–P). Moreover, the merged images of ECs and mCherry positive cells (Figs 2I–L,I′–L′ and 3I–L,I′–L′) demonstrated that the changes induced by CA-4 treatment of neuronal cells and blood vessels in trunk and brain were in the same tendency. The two-tailed Pearson correlation analyses demonstrated the numbers of mCherry positive cell and ISV lengths in trunk and brain were correlated with Pearson correlation coefficient 0.947 and 0.917, respectively (Figure S4A,B).

**Figure 5:**
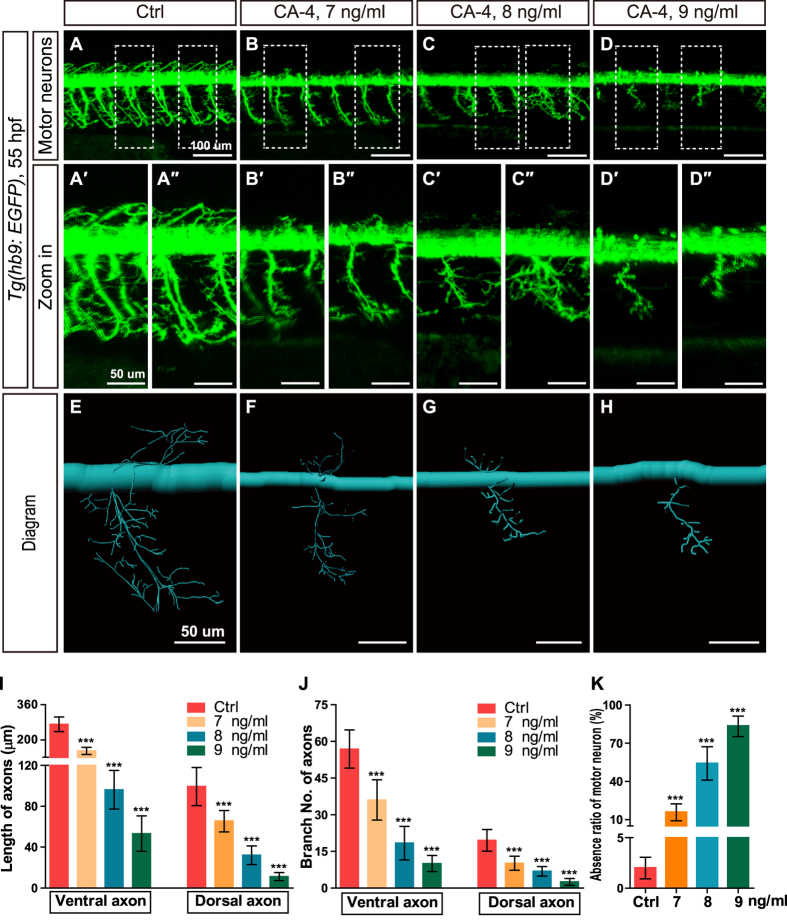
Effects of CA-4 treatment on motor neurons in the *Tg(hb9:EGFP)* embryonic zebrafish at 55 hpf. (**A–D**) Phenotype of motor neuron in control group and 7, 8, 9 ng/ml CA-4 treated groups at 55 hpf. Scale bar, 100 μm. (A′–D″) Zoomed in images of regions in dash line rectangles of panel A-D. Scale bar, 50 μm. (**E–H**) Diagrams of motor neurons. Scale bar, 50 μm. (**I–K**) Statistic analyses of the length and branch number of ventral and dorsal axons, as well as absence ratio of motor neurons in control group and 7, 8, 9 ng/ml CA-4 treated groups at 55 hpf. Data were expressed as the mean ± S.E.M. (n = 8). ***P < 0.001 vs. control group.

Next we investigated the effect of CA-4 treatment on motor neurons using transgenic line *Tg(hb9:EGFP)*, in which motor neurons are labeled with green florescence. It was shown that CA-4 treatment at 7–9 ng/ml significantly reduced the ventral and dorsal axon length and branch points of motor neurons (Fig. 5A–D,A′-D″), and CA-4 treatment at 8 and 9 ng/ml resulted in no apparent dorsal axon sprouting (Fig. 5C,D). The axons of motor neurons were diagrammed with Imaris software (Fig. 5E–H). Additionally, we found that CA-4 treatment also induced the absence of motor neurons (Fig. 5K). Through two-tailed Pearson correlation analysis, we found there were close correlations of axon length and ISV length, as well as absence ratio of motor neuron and absence ratio of ISV with Pearson correlation coefficient 0.895 and 0.903, respectively (Figure S4C,D).

### CA-4 treatment induced zebrafish neuronal apoptosis

**Figure 6:**
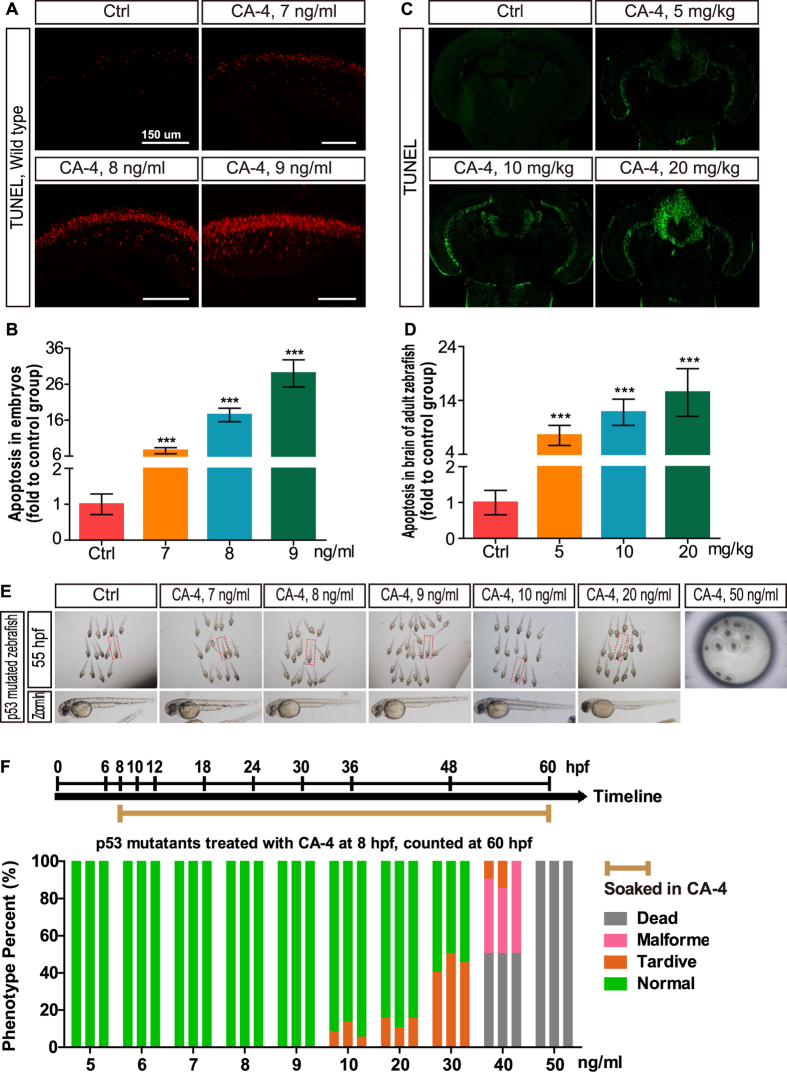
Cell apoptosis in central nervous system induced by CA-4 treatment. (**A,C**) Cell apoptosis in spinal cord of zebrafish embryos and brain section stained with TUNEL reagent of adult zebrafish. (**B,D**) Statistical analyses of cell apoptosis in central nervous system of embryonic and adult zebrafish. Data were expressed as mean ± S.E.M. (n = 4). ***P < 0.001 vs. control group. (**E**) The bright field images of p53 mutated embryonic zebrafish in control group and 7, 8, 9 ng/ml CA-4 treated groups at 55 hpf. (**F**) Percentage of phenotype in p53 mutants induced by 5–50 ng/ml CA-4 treatment at 8 hpf, analyzed at 60 hpf. The experiments of CA-4 treatment at each concentration were repeated in triplicate. The percentage of Dead, Malformed, Tardive and Normal was displayed in Grey, Pink, Orange and Green columns, respectively.

**Figure 7:**
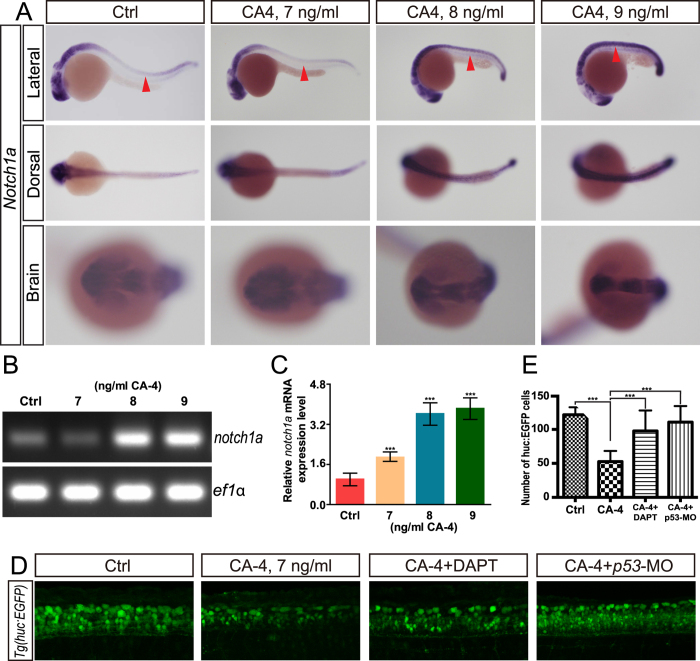
CA-4 treatment caused norch1a up regulation. (**A**) Whole-mount *in situ* hybridization analysis of notch1a expression in control embryo and CA-4-treated embryo. Red arrowhead indicates neural tube. (**B**) RT-PCR analysis of notch1a expression in control embryo and CA-4-treated embryo. (**C**) Real-time PCR analysis of notch1a expression in control embryo and CA-4-treated embryo. (**D**) Confocal analysis of CA-4-treated Tg (huc:EGFP) embryo. (**E**) Statistical analyses of EGFP positive cells in central nervous system of control, CA-4-treated, CA-4 and DAPT treated, and CA-4 treated p53 knockdown embryo. (n = 6), ***P < 0.001.

We found that CA-4 treatment caused spinal cord and brain region of zebrafish embryos become opaque (Figure S2), suggesting CA-4 treatment induces zebrafish neuronal apoptosis. To verify this hypothesis we did the TUNEL staining analysis of central neural system (CNS) in zebrafish embryos and adults treated with CA-4. It was shown that CA-4 treatment at 7–9 ng/ml induced obvious cell apoptosis at concentration dependent manner (Fig. 6A,B). Moreover, we found that the significant cell apoptosis in brain of adult zebrafish with 5–20 mg/kg CA-4 I.P. injection (Fig. 6C,D). To confirm CA-4 treatment induces zebrafish neuronal apoptosis, we treated the p53 mutated zebrafish embryos with different concentration of CA-4. It was revealed that CA-4 treatment at concentrations higher than 40 ng/ml led to embryos dead and the p53 mutants treated with CA-4 at concentrations lower than 9 ng/ml showed no obvious developmental defects, whereas CA-4 treatment at 7–9 ng/ml resulted in embryos developmental retardation or malformation (Fig. 6E,F). In addition, we treated the *p53* morpholino injected *Tg(huc:EGFP)* embryos with CA4 and found that p53 knockdown significantly reduced the apoptosis of EGFP positive cells (Fig. 7D). These results support that CA-4 treatment induces zebrafish neuronal apoptosis.

To investing the potential mechanism responsible for apoptosis, we examined the Notch and Wnt signaling in CA-4 treated zebrafish. A Wnt reporter *Tg(7xTCF-Xla.Siam:GFP)*^*ia4*^, which is reliable and sensitive Wnt biosensors for *in vivo* studies^19^,^20^, was used to evaluate the involvement of Wnt signaling in CA-4 induced neuronal apoptosis. We did not observe the obvious alteration of EGFP expression in central nervous system (CNS) of CA-treated embyos compared with that of control (Figure S5), indicating Wnt signaling is not responsible for the neuronal apoptosis. Then we examined the expression of Notch ligands and receptors using whole mount *in situ* hybridization analysis and found that *notch1a* was significantly up-regulated in CA-treated embyos (Figs 7A and S6A–F). This result was confirmed by real-time PCR and RT-PCR (Fig. 7B,C). It was reported that Notch activation induces apoptosis in neural progenitor cells through a p53-dependent pathway in mice^21^, which suggests that the elevation of *notch1a* expression linked to the neuronal apoptosis caused by CA-4 treatment. Inhibition of Notch signaling by DAPT partially rescued the apoptosis in zebrafish CNS caused by CA-4 (Fig. 7D), indicating that Notch signaling is involved in the neuronal apoptosis induced by CA-4. Additionally, we also examined the muscle structure in CA-4 treated embryos by Phalloidin staining, and did not detected apparent defects (Figure S7).

## Discussion

Humans are confronted with great stresses from environmental pollution, food safety, lifestyle shift and so on, resulting in a rising incidence of cancer diseases worldwide^22^,^23^. Based on the World Cancer Report 2014 of WHO, the worldwide burden of cancer is expected to rise to 22 million annually within the next two decades, and cancer deaths are predicted to rise from an estimated 8.2 million annually to 13 million per year^16^. The exploration of pathology mechanism of cancers and the development of therapeutic drugs are hot research topics in the field of medicine. Vigorous vasculature in tumor was commonly targeted in anti-tumor treatment. VDAs are the most important anti-tumor therapeutic agents. Among these agents, a large number of VDAs were designed and obtained based on the prototype compound CA-4. Thus, evaluating the efficacy and safety of CA-4 is necessary for the clinical application. In this study we firstly confirmed the anti-vascular effects of CA-4 using zebrafish model. Importantly, we found CA-4 displayed significant neuronal toxicity through inducing cell apoptosis in CNS.

Through investigation of dosing time window, we found CA-4 treatment from earlier stage caused zebrafish embryos more severe phenotype at the same dosage. And dosing from the same developmental stage, the embryos treated with CA-4 for longer duration displayed more severe phenotype. Dosing from 8 hpf and phenotyping at 36 hpf and 60 hpf, we demonstrated that CA-4 treatment at 4–10 ng/ml resulted in abnormal phenotype without death in wild type embryonic zebrafish. The previous studies reported 1 μmol/l CA4P (equivalent to 316 ng/ml CA-4) for 15 min obviously inhibited human lens epithelial cells^24^, and IC_50_ of 1.9–835 nmol/l CA-4 (equivalent to 0.6–263.84 ng/ml) showed against various human cancer cell lines and a MDR-resistant cancer cell line^6^. Compared with these reported dosages, our results showed the effective dosing range was relatively narrow, which might be attributed to the species diversity. In view of moderate efficacy, we selected 7–9 ng/ml CA-4 dosing at 8 hpf in the following work. 7–9 ng/ml CA-4 treatment induced ISVs and brain vessels inhibition or even absence in zebrafish embryos. Furthermore, we found CA-4 treatment inhibited ISV branching angiogenesis through retarding proliferation and migration of zebrafish ECs in dose-dependent manner. CA-4 and CA-4P were also reported to inhibit the proliferation and migration of epithelial cells *in vitro*^6^,^24^. Here we showed the potent anti-vascular effect of CA-4 via inhibiting proliferation and migration of developmental ECs *in situ*.

To investigate the anti-vascular effect of CA-4 in zebrafish adults, we conducted intraperitoneal injection of CA-4 into *Tg(fli1a:EGFP)* adult zebrafish. Thomas Nielsen *et al*. reported that 250 mg/kg CA-4P administered intraperitoneally significantly reduced tumor vessel volume and size distribution in mice^25^. It was also reported that rats dosed 30 and 100 mg/kg CA-4P showed tumor vascular shutdown^26^. 30 mg/kg CA-4P that Munich-Wistar rats received through intraperitoneal injection was considered to be a clinically relevant dose^27^,^28^,^29^,^30^. Based on these studies, we selected 5, 10 and 20 mg/kg as the treating dosages in the adult zebrafish experiments. Notably, intraperitoneal injection of 5 mg/kg CA-4, the lowest dosage we selected, resulted in potent anti-vascular effect in regenerated adult zebrafish pectoral fin. In conclusion, we firstly confirmed CA-4 possessed anti-vascular activity both in embryonic and adult zebrafish.

However, in the present study we reported firstly CA-4 exhibited potent negative effects in CNS of both embryonic and adult zebrafish. CA-4 treatment impairs zebrafish neural development and induces zebrafish neuronal apoptosis. CA-4 inducing apoptosis has been reported previously in several models *in vitro*^15^,^24^,^31^. The present data supports that CA-4 induces cell apoptosis *in vivo*. In addition, CA-4 has been reported to induce side effects such as anemia, dyspnoea, hypokalemia, headache, transient sensory neuropathy and renal impact^8^,^27^,^32^. In zebrafish embryos, CA-4 affected body formation, exerted potent anti-vascular effect, and resulted in abnormality in nervous system. These findings suggest that adequate evaluation on side effects and toxicity of CA-4 and its analogues is required in the future.

## Materials and Methods

### Ethics statement

All animal experimentation was carried out in accordance with the NIH Guidelines for the care and use of laboratory animals (http://oacu.od.nih.gov/regs/index.htm) and ethically approved by the Administration Committee of Experimental Animals, Jiangsu Province, China (Approval ID: SYXK(SU) 2007–0021).

### Zebrafish, drug treatment and morpholino injection

The study was conducted conforming to the local institutional laws, and the Chinese law for the Protection of Animals. The embryos were obtained through natural mating. Zebrafish embryos and adults were raised and maintained on standard conditions in Zebrafish Center of Nantong University as we previously described^33^,^34^. The transgenic zebrafish lines *Tg(kdrl:EGFP)*, *Tg(kdrl:EGFP::huc:mCherry)*, *Tg(fli1a:nEGFP)*, *Tg(hb9:EGFP)* and *p53* mutants were used as described in previous work^34^,^35^,^36^. Developmental stages of embryonic zebrafish referred to the previously described by Kimmel *et al*.^37^. At 6 hpf, embryos were screened under anatomical microscope to remove the morphologically abnormal individuals. Around 10 healthy embryos were loaded into each well of 96-well plate in E3 solution. At the setting time, E3 solutions were replaced with CA-4 treatment solutions. The control and treated groups were analyzed at different intervals. At 55 hpf, zebrafish embryos were collected for imaging and fixed with 4% paraformaldehyde (PFA) in phosphate-buffered saline (PBS) for TUNEL staining.

Adult *Tg*(*fli1a*:*EGFP*) zebrafish line at 6 months post fertilization were randomly divided into control group (n = 10, intraperitoneal injection normal saline), 5, 10 and 20 mg/kg groups (n = 10, intraperitoneal injection CA-4 solution). Zebrafish was anesthetized with 0.16-mg/ml tricaine and one of the pectoral fins was amputated at the first injection. Confocal imaging analysis of pectoral fins was carried out at 3 and 9 days post amputation (dpa) respectively. After intraperitoneal injection daily for 10 days, all the zebrafish were sacrificed and the brain tissues were collected for TUNEL staining. The DAPT treatment and morpholino injection was carried out as previously described^38^,^39^.

### Cryostat section and TUNEL staining

Zebrafish embryos or tissues were fixed with 4% PFA overnight at 4 °C. Then samples were washed for 3 × 5 min with PBS and immersed with the melted agarose-sucrose (1.5% agarose, 5% sucrose). The solidified agarose block was trimmed and incubated in 30% sucrose overnight at 4 °C. After being embedded with OCT compound (Tissue Tek), the block was fixed on the platform and equilibrated in the chamber of cryostats at least 30 min, and then sectioned at 10 μm according to the manufacture’s instruction. We mounted the sections on slides and dried the slides in the air for 4–5 hours. The sections were re-fixed on the slides for 20–30 min with 4% PFA, washed with PBS 3 × 5 min and incubated in permeabilisation solution (0.1% Triton X-100, 0.1% sodium citrate) for 2 min on ice. The TUNEL reaction solution was prepared according to the manufacture’s instruction (Roche). The sections were labeled with TUNEL reaction solution strictly following the manufacture’s protocol (Roche).

### RNA extraction, reverse transcription, and PCR

Tissue was homogenized and frozen in Trizol reagent (Invitrogen) and stored at −80 °C. Total RNA was extracted following the manufacturer’s instructions. 1 μg of RNA was reverse transcribed into cDNA using Transcriptor First Strand cDNA Synthesis Kit (Roche) according to the manufacturer’s instructions. Synthesized cDNA was stored at −20 °C. The Left primer for RT-PCR is 5′-ATGACATCACCCTTCCAGCA-3′; Right primer is 5′-GGTGATTGGGTGTGTTGTCC-3′. PCR amplifications were carried out in a total volume of 50 μl using specific primers and Advantage2 Polymerase Kit (Clontech). The Left primer for Real-time PCR is 5′- ATTGATGAGTGTGTGAGCGC-3′; Right primer is 5′- CAGTTGATGCCACTGAAGCC-3′. The Real-time PCR was carried out as previously described^38^.

### Riboprobe synthesis and whole-mount *in situ* hybridization

The coding sequence for zebrafish Notch signaling genes were amplified by PCR using the primers as previously described^40^. Digoxigenin (DIG)-labeled RNA sense and antisense probes were made from the linearized plasmids according to the manufacturer’s protocol using the DIG RNA Labeling Kit (SP6/T7) (Roche). Whole-mount *in situ* hybridization was carried out as we previously described^35^,^41^.

### Imaging

At 55 hpf, for confocal imaging embryos were anesthetized with E3/0.16 mg/mL tricaine/1% 1-phenyl-2-thiourea (Sigma) and embedded in 0.8% low melt agarose. Images were acquired with an Olympus DP71 camera on an Olympus stereomicroscope. Confocal imaging was performed with a Leica TCS-SP5 LSM. Analysis was performed using Imaris software. The final figure processing was performed with Adobe Photoshop and Illustrator CS6.

### Statistics

ISV length, size of regenerated pectoral fin vascular plexus, mCherry positive cell number, motor neuron axon length, and branch points were measured with Imaris (version 7.2.3). These data were statistically analyzed with GraphPad Prism 5. All data were expressed as mean ± S.E.M. Statistical analysis were performed using a one-way analysis of variance (ANOVA) (P < 0.05). The correlations between the changes of quantifications of blood vessels and those of nervous systems were analyzed by SPSS software (version 13.0).

## Additional Information

**How to cite this article**: Shi, Y.-W. *et al*. Combretastatin A-4 efficiently inhibits angiogenesis and induces neuronal apoptosis in zebrafish. *Sci. Rep.* **6**, 30189; doi: 10.1038/srep30189 (2016).

## Supplementary Material

Supplementary Information
