# Supplementary material for: Combretastatin A-4 efficiently inhibits angiogenesis and induces neuronal apoptosis in zebrafish
Source: Sci Rep. 2016 Jul 25;6:30189. doi: 10.1038/srep30189 (PMC4958954; doi:10.1038/srep30189)
Supplement: Supplementary Information [file srep30189-s1.pdf]

Title: Combretastatin A-4 efficiently inhibits angiogenesis and induces neuronal apoptosis in zebrafish

Running title: CA-4 inhibits angiogenesis and induces neuronal apoptosis

Yun-Wei Shi[1, #, *], Wei Yuan[1, #], Xin Wang[1], Jie Gong[2], Shun-Xing Zhu[3], Lin-Lin Chai[1], Jia-Ling Qi[4], Yin-Yin Qin[1], Yu Gao[1], Yu-Ling Zhou[4], Xiao-Le Fan[4], Chun-Ya Ji[4], Jia-Yi Wu[4], Zhi-Wei Wang [1, 5], Dong Liu[1, *]

1, *Co-innovation Center of Neuroregeneration, Jiangsu Key Laboratory of Neuroregeneration, Nantong University, Nantong, Jiangsu 226001, PRC*

2, *School of medicine, Nantong University, Nantong, Jiangsu 226001, PRC*

3, *School of life science, Nantong University, Nantong, Jiangsu 226001, PRC*

4, *Laboratory Animal Center, Nantong University, Nantong, Jiangsu 226001, PRC*

5, *Department of Pharmacology, University of California, Irvine, CA 92697, USA*

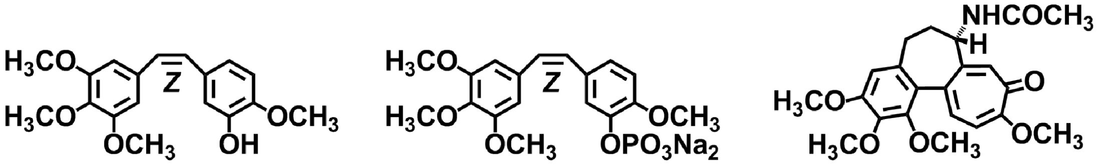

cis-Combretastatin A-4 (CA-4) [CAS, 117048-59-6]

cis-Combretastatin A-4 disodium phosphate (CA-4P) [CAS, 168555-66-6]

Colchicine [CAS, 64-86-8]

Figure S1. Chemical structures of CA-4, CA-4P and colchicine.

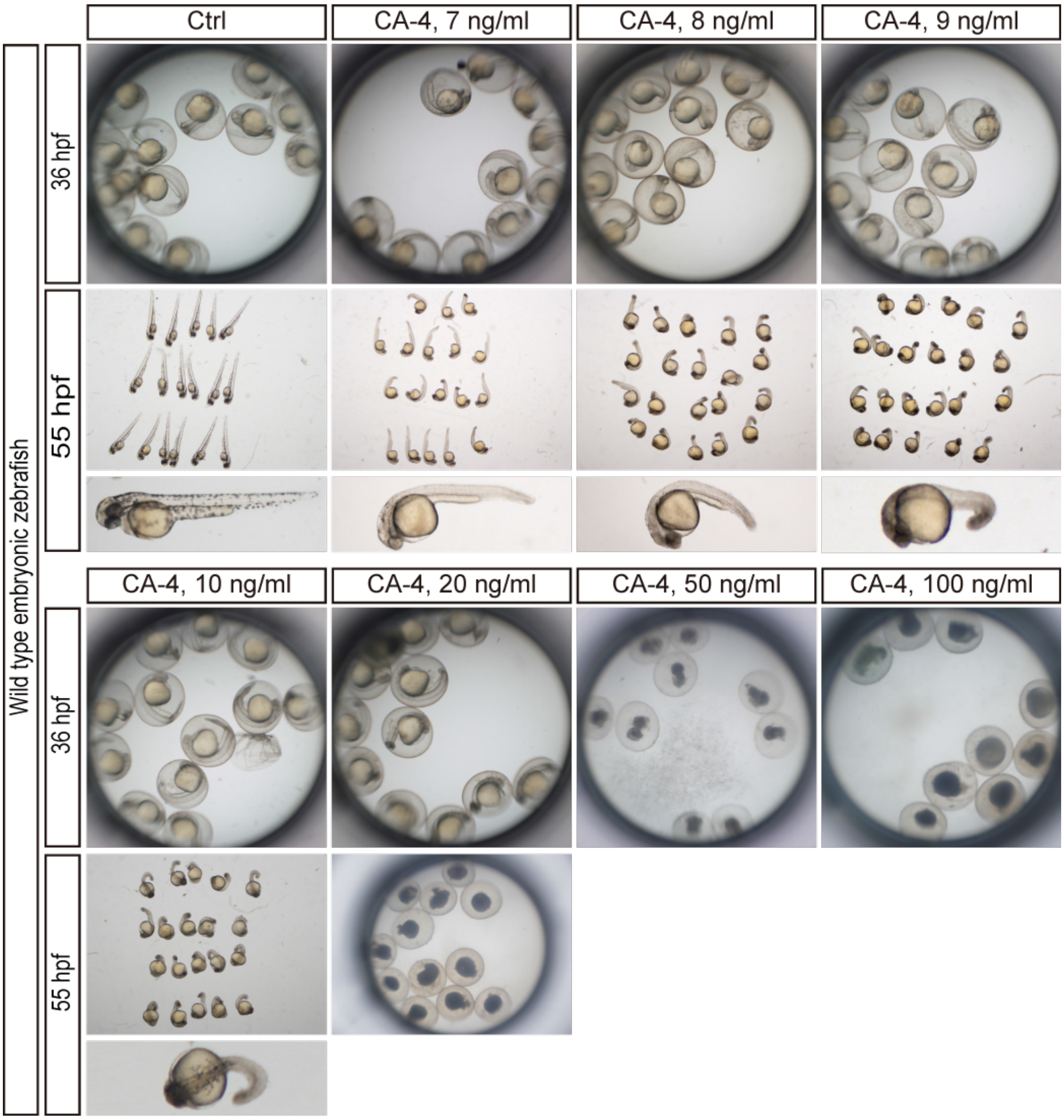

Figure S2. Phenotype of embryonic zebrafish induced by CA-4 treatment at 36 hpf and 60 hpf. The experiments of CA-4 treatment at each concentration were repeated in triplicate.

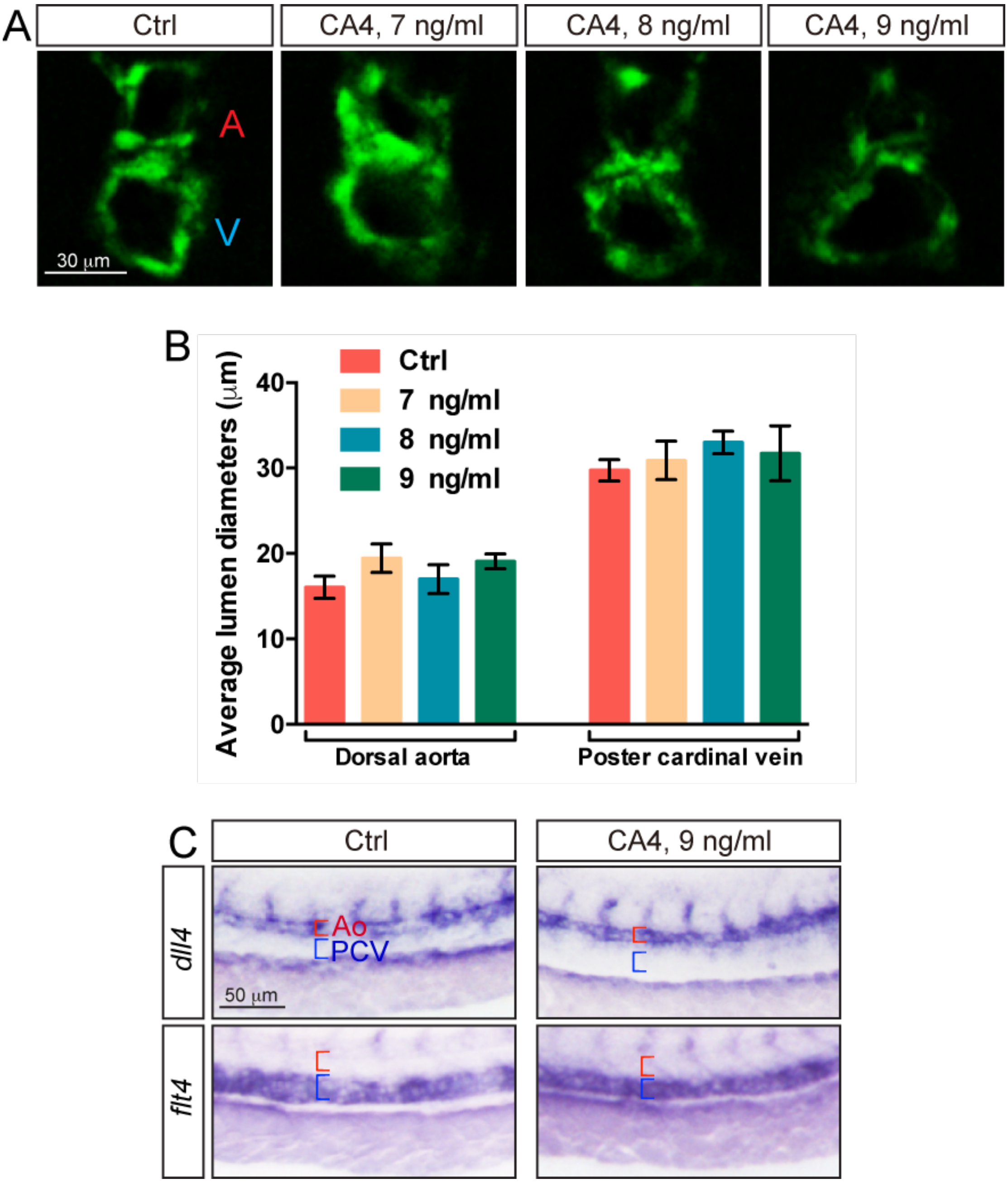

Figure S3. The lumen size of DA and PCV were not significantly changed in CA-4 treated zebrafish embryos. (A) Cross-section of *Tg(kdrl:EGFP)* control zebrafish embryos and embryos treated with CA-4 at different concentrations. (B) The statistics of DA and PCV lumen size, error bars indicate s.e.m. (20 embryos were analyzed for each group). (C) Whole-mount *in situ* hybridization analysis in 30 hpf control and CA-4 treated zebrafish embryos using *flt4* (n=12) and *dll4* (n=15) antisense probes.

A

		mcherry + Cell No.	ISV Length
mcherry + Cell No.	Pearson Correlation	1	.947**
	Sig. (2-tailed)		.000
	N	32	32
ISV Length	Pearson Correlation	.947**	1
	Sig. (2-tailed)	.000	
	N	32	32

**. Correlation is significant at the 0.01 level (2-tailed).

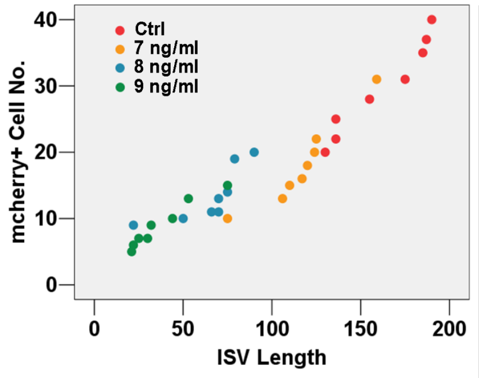

B

		Brain Vessels	mcherry + Cell No.
Brain Vessels	Pearson Correlation	1	.917**
	Sig. (2-tailed)		.000
	N	32	32
mcherry + Cell No.	Pearson Correlation	.917**	1
	Sig. (2-tailed)	.000	
	N	32	32

**. Correlation is significant at the 0.01 level (2-tailed).

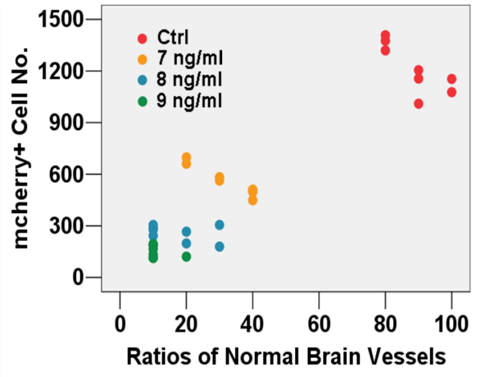

C

		Motor Neuron Axon Length	ISV Length
Motor Neuron Axon Length	Pearson Correlation	1	.895**
	Sig. (2-tailed)		.000
	N	32	32
ISV Length	Pearson Correlation	.895**	1
	Sig. (2-tailed)	.000	
	N	32	32

**. Correlation is significant at the 0.01 level (2-tailed).

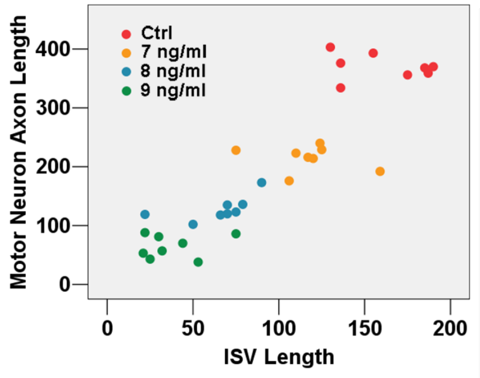

D

		Absence Rate of ISVs	Absence Rate of Motor Neurons
Absence Rate of ISVs	Pearson Correlation	1	.903**
	Sig. (2-tailed)		.000
	N	32	32
Absence Rate of Motor Neurons	Pearson Correlation	.903**	1
	Sig. (2-tailed)	.000	
	N	32	32

**.Correlation is significant at the 0.01 level (2-tailed).

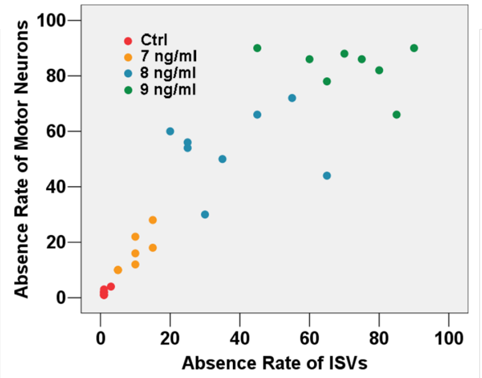

Figure S4. Two-tailed pearson correlation analyses. (A) Number of neuronal precursor cell in spinal cord *vs* ISV length, (B) Number of neuronal precursor cells in brain vs that of brain vessels, (C) motor neuron axonal length vs ISV length, and (D) absence ratio of motor neuron vs absence ratio of ISV.

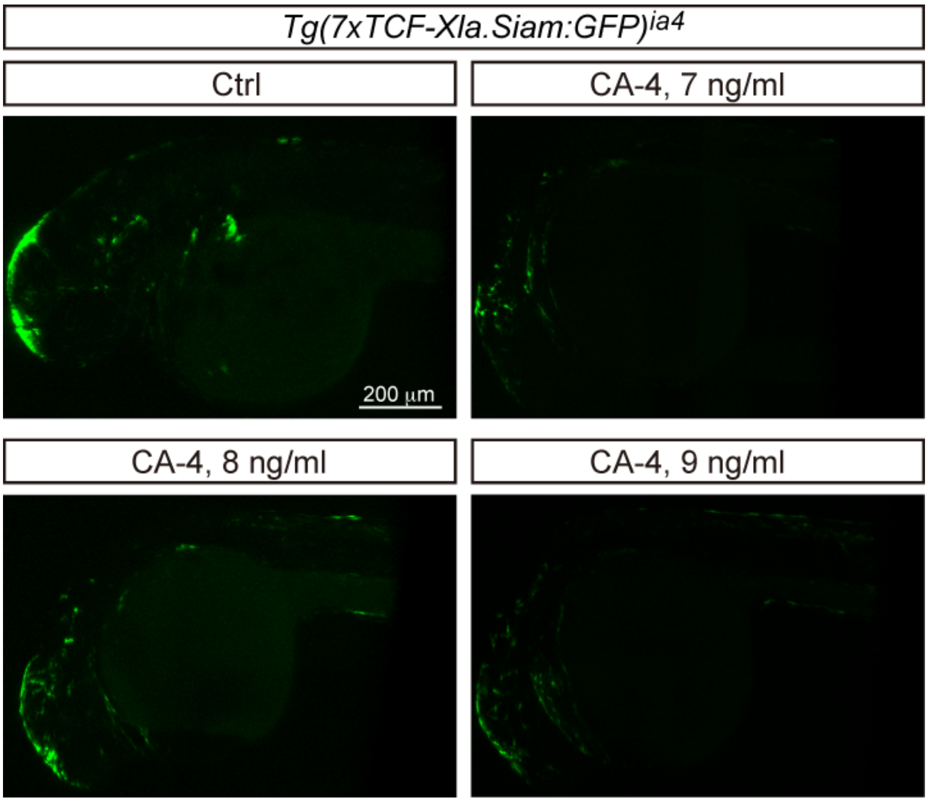

Figure S5. Confocal analysis of EGFP expression in CA-4 treated *Tg(7xTCF-Xla.Siam:GFP)*^{ia4} embryos.

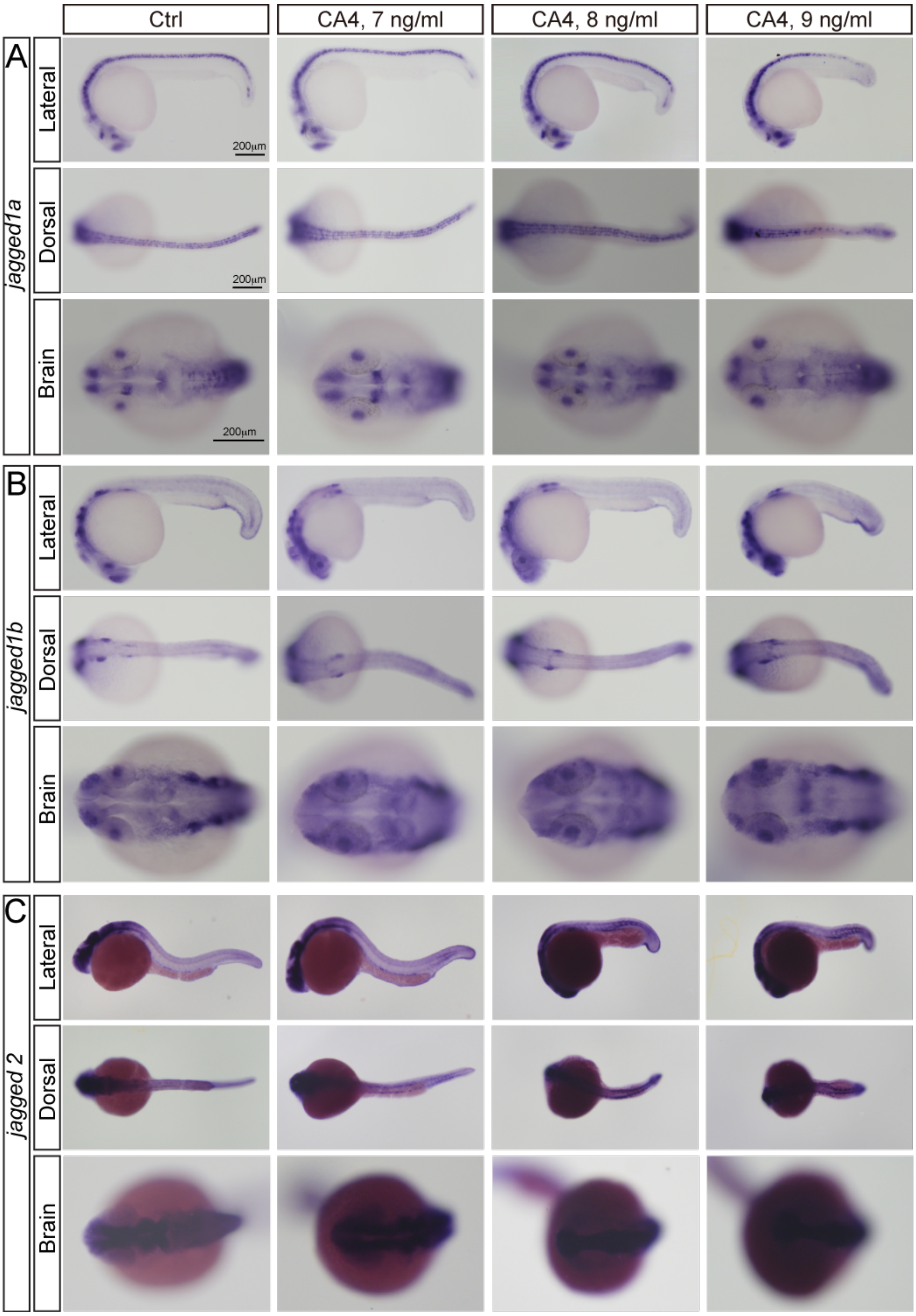

To be continued

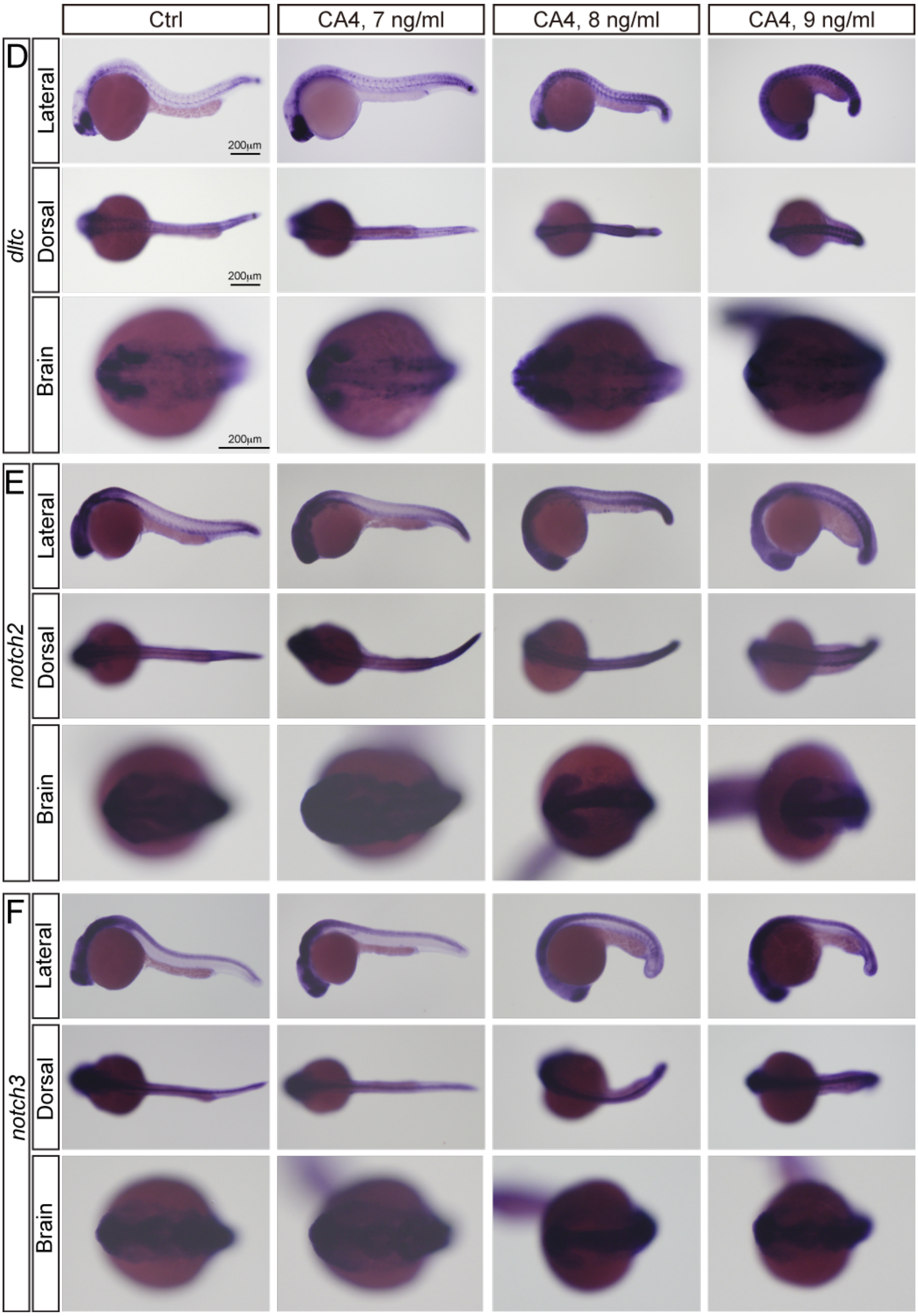

Figure S6 Whole- mount *in situ* hybridization analysis of *jaggged1a*, *jagged1b*, *jagged2, dltc*, *notch2* and *notch3* expression in control embryo and CA-4- treated embryo.

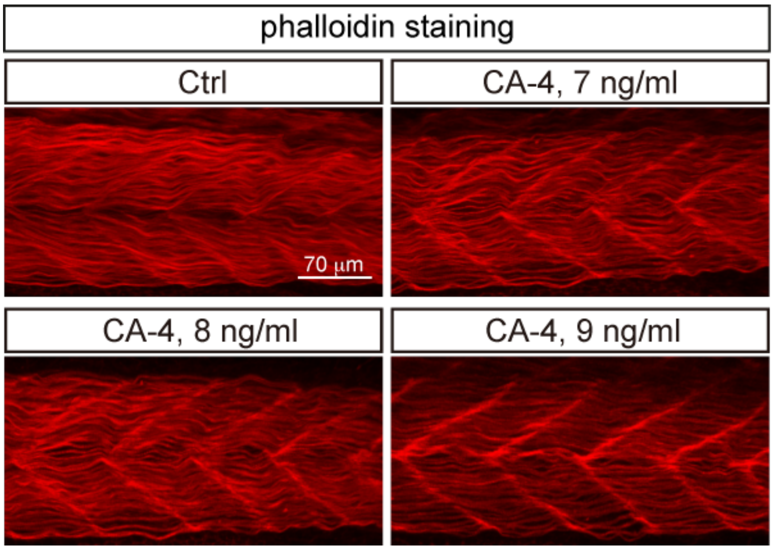

Figure S7 Confocal imaging analysis of zebrafish somites of CA-4 treated embryos stained with Phalloidin, showing the muscle fibers.
